# Diesel exhaust particulate induces pulmonary and systemic inflammation in rats without impairing endothelial function *ex vivo *or *in vivo*

**DOI:** 10.1186/1743-8977-9-9

**Published:** 2012-04-05

**Authors:** Sarah Robertson, Gillian A Gray, Rodger Duffin, Steven G McLean, Catherine A Shaw, Patrick WF Hadoke, David E Newby, Mark R Miller

**Affiliations:** 1 Centre of Cardiovascular Science, University of Edinburgh, Edinburgh, Scotland, UK; 2 Centre for Inflammation Research, University of Edinburgh, Edinburgh, Scotland, UK; 3 BHF/University Centre for Cardiovascular Science, University of Edinburgh, The Queen's Medical Research Institute, 47 Little France Crescent, Edinburgh EH16 4TJ, UK

**Keywords:** Diesel, Pollution, Particle, Particulate, Blood vessel, Artery, Vasodilatation, Endothelium, Inflammation

## Abstract

**Background:**

Inhalation of diesel exhaust impairs vascular function in man, by a mechanism that has yet to be fully established. We hypothesised that pulmonary exposure to diesel exhaust particles (DEP) would cause endothelial dysfunction in rats as a consequence of pulmonary and systemic inflammation.

**Methods:**

Wistar rats were exposed to DEP (0.5 mg) or saline vehicle by intratracheal instillation and hind-limb blood flow, blood pressure and heart rate were monitored *in situ *6 or 24 h after exposure. Vascular function was tested by administration of the endothelium-dependent vasodilator acetylcholine (ACh) and the endothelium-independent vasodilator sodium nitroprusside (SNP) *in vivo *and *ex vivo *in isolated rings of thoracic aorta, femoral and mesenteric artery from DEP exposed rats. Bronchoalveolar lavage fluid (BALF) and blood plasma were collected to assess pulmonary (cell differentials, protein levels & interleukin-6 (IL-6)) and systemic (IL-6), tumour necrosis factor alpha (TNFα) and C-reactive protein (CRP)) inflammation, respectively.

**Results:**

DEP instillation increased cell counts, total protein and IL-6 in BALF 6 h after exposure, while levels of IL-6 and TNFα were only raised in blood 24 h after DEP exposure. DEP had no effect on the increased hind-limb blood flow induced by ACh *in vivo *at 6 or 24 h. However, responses to SNP were impaired at both time points. In contrast, *ex vivo *responses to ACh and SNP were unaltered in arteries isolated from rats exposed to DEP.

**Conclusions:**

Exposure of rats to DEP induces both pulmonary and systemic inflammation, but does not modify endothelium-dependent vasodilatation. Other mechanisms *in vivo *limit dilator responses to SNP and these require further investigation.

## Background and objectives

Exposure to air pollution has been associated with increased cardiovascular mortality and morbidity [1-3]. These associations are strongest for the particulate matter (PM) in air pollution, and the World Health Organisation has estimated that airborne particles are responsible for half a million premature deaths each year [4]. Ultrafine particles (or nanoparticles) are of specific concern because their small size allows them to penetrate deep into the respiratory tract [5] and also engenders them with a large reactive surface area. Exhaust from diesel engines is especially rich in nanoparticles and, therefore, may contribute greatly to the health effects of PM in urban environments [6,7].

The mechanism(s) by which inhaled PM alters cardiovascular function has not been established. We have shown that controlled exposure to diesel exhaust impairs endothelial vasomotor function in healthy volunteers [8-10] and in patients with stable coronary heart disease [11]. The vascular impairment observed appears to be mediated by the particulate component of the exhaust rather than the gaseous co-pollutants [7,10]. Furthermore, *ex viv*o exposure of blood vessels to diesel exhaust particles (DEP) inhibits nitric oxide (NO)-mediated vasodilatation via generation of superoxide free radicals [12]. Thus, DEP can directly alter endothelial cell function but this assumes that a considerable number of the particles are able to translocate from the lung to the circulation. While studies have demonstrated that translocation of nanoparticles is feasible [13-15] there remains considerable uncertainty over whether this mechanism underlies the health effects of combustion-derived nanoparticles [16-18]. An alternative suggestion is that inflammation induced by PM in the lung may spill-over into the systemic circulation, causing indirect cardiovascular changes [19]. Several different types of particulate have been shown to induce pulmonary inflammation [18,20], but the occurrence and potential role of systemic inflammation following pulmonary exposure to particulates is often inconsistent [8,21,22]. We hypothesise that instillation of DEP will cause endothelial dysfunction in rats as a consequence of pulmonary and systemic inflammation. Analysis of arteries isolated from PM-exposed animals has generally shown little evidence of dysfunction. However, this may be due to limitations of *ex vivo *analyses, which remove the vessel from neurohumoural control *in vivo*. Therefore, we addressed our hypothesis by measuring arterial function both *in vivo *(in the hind-limb resistance bed) and *ex vivo *in isolated conduit (aorta, femoral) and resistance (mesenteric) arteries following intra-tracheal instillation of rats with DEP or vehicle (saline).

## Results

### Assessment of pulmonary inflammation

**Figure 1:**
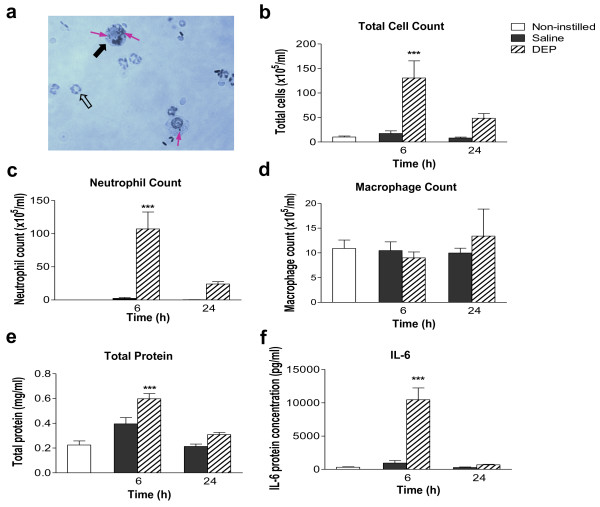
**Instillation of diesel exhaust particles (DEP) causes transient pulmonary inflammation**. **(a) **Representative photomicrograph of a cytocentrifuge slide prepared from bronchoalveolar lavage fluid (BALF) collected from a DEP-instilled animal 6 h after instillation. Neutrophils (open arrow) and alveolar macrophages (black arrow) containing particulate (purple arrow) are apparent. Diff-Quick™ staining, ×400 magnification. BALF was analysed for **(b) **total cell count, **(c) **neutrophils, **(d) **alveolar macrophages, **(e) **total protein (bicinchonic acid assay) and **(f) **interleukin-6 (IL-6). Non-instilled (open columns), saline-instilled (solid columns) and DEP-instilled (hatched columns) animals 6 and 24 h after instillation. Results are expressed as mean ± SEM (n = 4-8; data for non-instilled groups were pooled, n = 11) ***P < 0.001 DEP versus saline; two-way ANOVA followed by Bonferroni post-hoc test.

Instillation of DEP was associated with an influx of neutrophils and macrophages into bronchoaveolar lavage fluid (BALF). Black particles were evident within these cells following DEP instillation (Figure 1a). Instillation of saline produced no significant alteration in the total cell number in BALF compared with untreated control animals. However, instillation of DEP increased the number of cells in lavage 6 h and 24 h post-exposure (Figure 1b). Total cell counts were greatest 6 h post-exposure (130 ± 35 × 10^5^/mL versus 17 ± 5 × 10^5^/mL in saline controls, *P *< 0.001) and remained elevated at 24 h (49 ± 10 × 10^5 ^cells/mL versus 8.3 ± 1.8 × 10^5 ^cells/mL in saline-treated controls). The increase in the total cell count 6 h after DEP instillation was predominately due to increases in neutrophil number (Figure 1c). There were no differences in the number of macrophages in BALF between the treatment groups (Figure 1d) and, in all groups, eosinophil and lymphocyte numbers were below the threshold for detection. This pattern of cell differentials was identical when expressed as a percentage of total cell number (data not shown).

Protein concentration in the BALF was measured as a surrogate of lung permeability [23]. After saline-instillation, total protein concentration was similar to baseline levels at all time-points (Figure 1e). In contrast, total protein increased 2- to 3-fold 6 h after DEP instillation (*P *< 0.001, n = 6-8; Figure 1e), with levels returning to baseline by 24 h. Measurement of the cytokine interleukin-6 (IL-6) showed a similar pattern with levels increasing above baseline (0.30 ± 0.07 ng/ml) 6 h after DEP instillation (10.5 ± 1.8 ng/ml; *P *< 0.001) and returning to baseline within 24 h (Figure 1f). Levels of tumour necrosis factor alpha (TNFα) and C-reactive protein (CRP) were also measured in the BALF, but both were below the limits of detection (< 16 pg/ml for TNFα and < 39 pg/ml for CRP).

### Assessment of inflammatory response in blood

**Figure 2:**
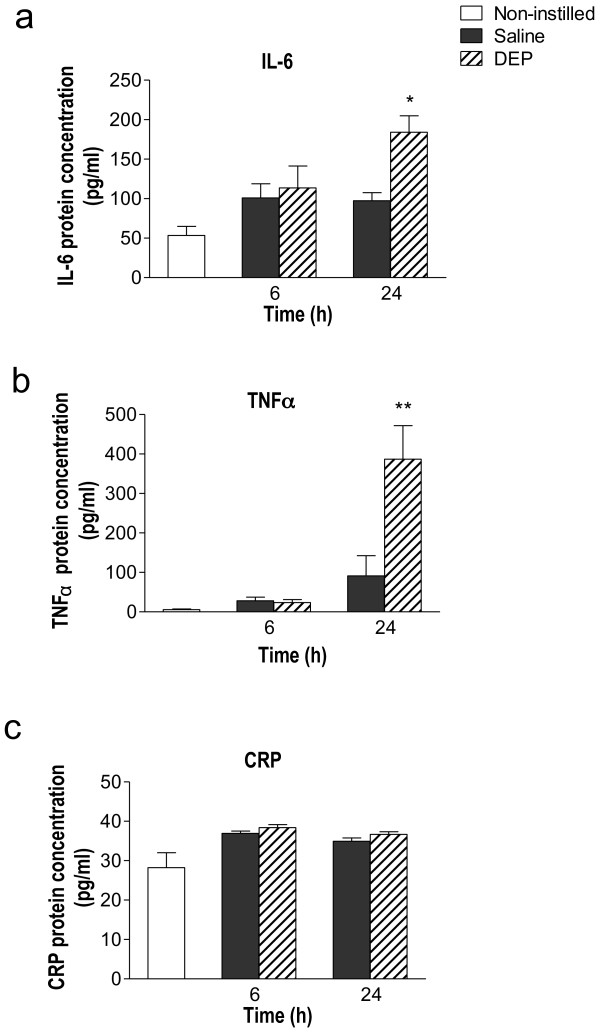
**Instillation of diesel exhaust particles (DEP) causes systemic inflammation**. Plasma cytokines **(a) **Interleukin-6 (IL-6) **(b) **Tumour necrosis factor alpha (TNFα) &** (c) **C-reactive protein (CRP) were detected by ELISA. Non-instilled (open columns), saline-instilled (solid column) or DEP-instilled (0.5 mg; hatched columns) animals 6 and 24 h after instillation. Results are expressed as mean ± SEM (n = 4-6). , *P < 0.05, **P < 0.01 DEP versus saline; two-way ANOVA followed by Bonferroni post-hoc test.

Red blood cell and platelet counts were not different across treatment groups (Table 1). There was a significant increase in numbers of white blood cells at 6 h after instillation (*P *< 0.05), that decreased to levels that were not significantly different to those of the saline-treated group at 24 h (Table 1). Baseline plasma IL-6 and TNFα levels were 53.5 ± 11.5 pg/ml and 5.8 ± 1.5 pg/ml, respectively (Figure 2a &2b) and there was no significant change from baseline at 6 h after DEP or saline instillation. At 24 h after instillation, DEP induced significantly higher levels of both IL-6 (*P *< 0.05; Figure 2a) and TNFα (*P *< 0.01; Figure 2b) in comparison to the saline instilled group. Although intratracheal instillation of DEP elevated baseline levels of circulating CRP at both time points (Figure 2c), no statistical differences were observed between the saline and DEP groups.

**Table 1:** Blood cell differentials 6 or 24 h after instillation of diesel exhaust particulate (DEP) or saline

	Treatment group				
	
	6 h			24 h	
	
	Non-instilled	Saline	DEP	Saline	DEP
**RBC** (×10^3^/pl)	5.9 ± 0.3	5.4 ± 0.3	5.6 ± 0.2	5.6 ± 0.3	5.3 ± 0.3
**WBC** (×10^3^/μl)	4.6 ± 0.5	5.8 ± 0.4	8.6 ± 0.3*	4.6 ± 1.0	5.7 ± 1.4
**platelets** (×10^3^/μl)	358 ± 93	415 ± 106	447 ± 49	525 ± 177	618 ± 164

Abbreviations: DEP, diesel exhaust particles; RBC = red blood cells; WBC, white blood cells.

Results are mean ± SEM (n = 5). Not significant (P > 0.05) unless otherwise stated. *P < 0.05, saline vs DEP, Bonferroni post-hoc test, following two-way ANOVA

### *In vivo *vascular function

Baseline systolic blood pressure was significantly higher in DEP-, than in saline-, treated rats 6 h after exposure (*P *< 0.05; Table 2). Diastolic and mean arterial pressure showed the same trend, but this failed to reach statistical significance. Baseline heart rate and blood flow were not different between treatment groups (Table 2).

**Table 2:** Baseline heart rate, arterial pressure and hind-limb blood flow 6 and 24 h after instillation of diesel exhaust particulate (DEP) or saline (assessed before vasodilator administration)

	Treatment group				
	
	6 h			24 h	
	
	Non-instilled	Saline	DEP	Saline	DEP
**HR** (bpm)	354 ± 6	352 ± 7	372 ± 11	335 ± 6	353 ± 7
**SBP** (mmHg)	103.9 ± 5.7	105.1 ± 5.1	130.7 ± 13.4*	101.3 ± 2.4	116.9 ± 5.9
**DBP** (mmHg)	88.9 ± 6.5	98.7 ± 5.0	122.5 ± 12.8	85.3 ± 2.2	101.8 ± 7.1
**MABP** (mmHg)	91.4 ± 7.4	100.9 ± 5.0	125.3 ± 13.0	91.2 ± 1.8	106.8 ± 6.5
**HBF** (ml/min)	2.3 ± 0.3	2.4 ± 0.4	2.7 ± 0.2	2.0 ± 0.3	2.6 ± 0.3

DEP, Diesel Exhaust Particles; HR, heart rate; SBP, systolic blood pressure; DBP, diastolic blood pressure; MABP, mean arterial blood pressure; HBF, hind-limb blood flow.

Results are mean ± SEM (n = 5-8). Not significant (*P *> 0.05) unless otherwise stated. *P < 0.05, saline vs DEP, Bonferroni post-hoc test, following two-way ANOVA

**Figure 3:**
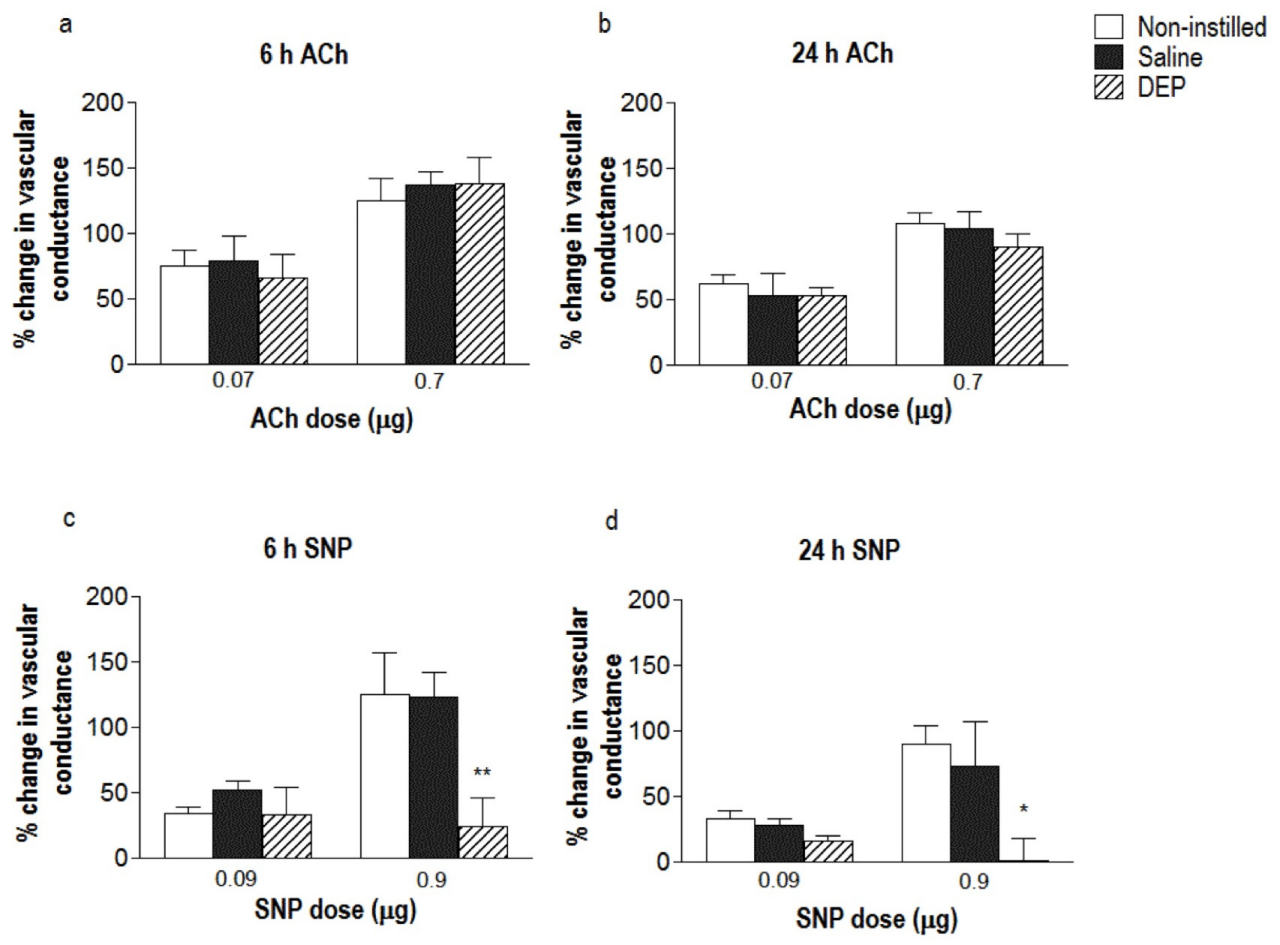
**Instillation of diesel exhaust particles (DEP) does not affect endothelium-dependent relaxation, but impairs vasodilator response to sodium nitroprusside, *in vivo***. Percent change in femoral vascular conductance (FVC) from baseline in response to intra-arterial acetylcholine (ACh; 0.07 & 0.7 μg) and sodium nitroprusside (SNP; 0.09 & 0.9 μg) 6 h **(a **&**c) **and 24 h **(b **&**d) **after instillation. Responses were obtained in non-instilled rats (open column), saline-instilled rats (solid column) or DEP-instilled rats (0.5 mg; hatched column). Results are expressed as mean ± SEM (n = 4-6). *P < 0.05, **P < 0.01 DEP versus saline; two-way ANOVA followed by Bonferroni post-hoc test.

Intra-arterial injections of acetylcholine (ACh) into the hind-limb vascular bed increased femoral vascular conductance (FVC; Figure 3a &3b) without affecting mean arterial blood pressure. Neither saline nor DEP administration altered arteriolar dilations in response to ACh at either 6 h (Figure 3a) or 24 h (Figure 3b) after instillation. Intra-arterial administration of sodium nitroprusside (SNP) was associated with a tendency for a transient reduction in mean arterial blood pressure (6-12 mmHg in both saline- and DEP- exposed rats) but this change in blood pressure was not statistically significant in any group (*P *> 0.10 for all). SNP (0.9 μg) produced approximately a 2-fold increase in vascular conductance above baseline in the non-instilled control animals and in saline-instilled animals at 6 h or 24 h after instillation (Figure 3c & Figure 3d). At both time points DEP significantly suppressed this increase in conductance evoked by SNP (Figure 3c &3d).

### *Ex vivo *vascular function

**Figure 4:**
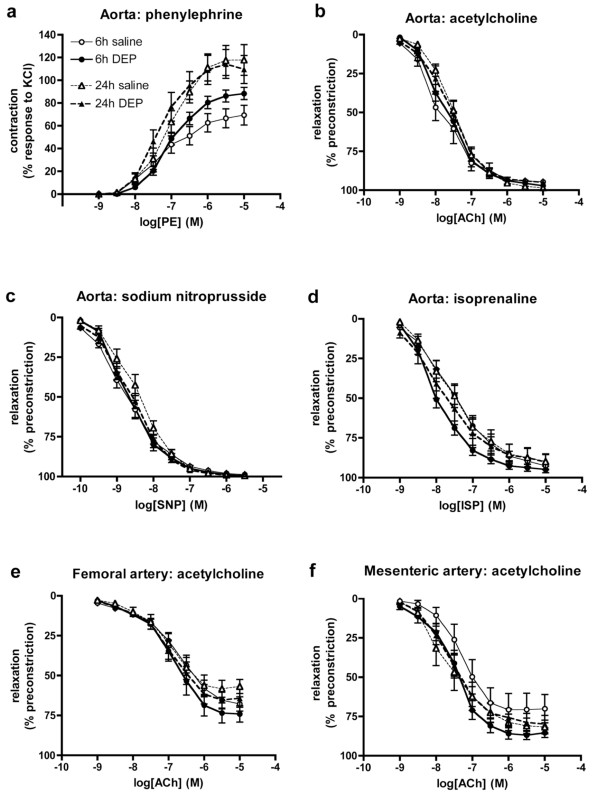
**Instillation of diesel exhaust particles (DEP) has no effect on vascular responses *ex vivo***. **(a) **Contraction to phenylephrine (PE), and relaxation to **(b) **acetylcholine (ACh), **(c) **sodium nitroprusside (SNP) and **(d) **isoprenaline (ISP) in the thoracic aorta. Responses to ACh in **(e) **femoral artery and **(f) **3^rd ^order mesenteric arteries. Data from saline-instilled animals (open symbols) and DEP-instilled animals (closed symbols) sacrificed at 6 h (circles, solid line) or 24 h (triangles, dashed line) after instillation. Results are expressed as mean ± SEM (n = 6-10). No significant differences were found between saline or DEP instilled animals at either time points for all vessels (two-way ANOVA).

Responses to vasodilator drugs were assessed *ex vivo *in thoracic aortas from rats, 6 or 24 h after instillation (Figure 4, Table 3). DEP-instillation had no effect on the sensitivity of the contractile responses to phenylephrine in either the 6 h (*P *= 0.31) or 24 h groups (*P *= 0.82; Figure 4a), although there was a significantly greater maximum contraction to phenylephrine in the 6 h DEP group (*P *= 0.045; Table 3). Importantly, DEP had no influence on vasodilator responses to ACh (*P *= 0.68 for 6 h, *P *= 0.89 for 24 h; Figure 4b) or SNP (*P *= 0.79 for 6 h, *P *= 0.22 for 24 h; Figure 4c) at either time point. In an additional group of animals sacrificed 2 h after instillation, responses to ACh and SNP were identical in saline- and DEP-treated animals at this earlier time point (n = 6; data not shown). There was no significant difference between the concentration-response curves to isoprenaline, as a whole, in the aortas from saline- and DEP-treated animals (two-way ANOVA; *P *> 0.06; Figure 4d), although a small, but significant, difference in IC_50 _values was observed at 6 h (unpaired t-test; *P *= 0.013; Table 3).

**Table 3:** *Ex vivo *contractile and relaxant responses in arterial rings isolated 6 and 24 h after instillation of rats with diesel exhaust particles (DEP) or saline

	phenylephrine		acetylcholine		sodium nitroprusside		isoprenaline	
	logEC_50_	Max (% response to KCl)	logIC_50_	Imax (% preconstriction)	logIC_50_	Imax (% preconstriction)	logIC_50_	Imax (% preconstriction)
**Aorta**								
**6 h**								
saline	-7.24 ± 0.14	**67.1 ± 8.1**	-7.88 ± 0.20	94.9 ± 1.3	-8.67 ± 0.13	96.7 ± 1.0	**-7.52 ± 0.15**	90.0 ± 1.8
DEP	-7.03 ± 0.07	88.6 ± 5.1*	-7.75 ± 0.11	96.1 ± 1.4	-8.70 ± 0.11	87.9 ± 1.0	-8.07 ± 0.10*	93.1 ± 2.2
**24 h**								
saline	-7.29 ± 0.13	120.8 ± 14.5	-7.52 ± 0.10	99.3 ± 0.5	-8.43 ± 0.13	99.3 ± 0.6	-7.62 ± 0.16	88.0 ± 5.8
DEP	-7.38 ± 0.11	115.3 ± 13.0	-7.67 ± 0.17	96.2 ± 1.5	-8.60 ± 0.10	98.9 ± 0.4	-7.62 ± 0.26	87.8 ± 6.4
**Femoral Artery**								
**6 h**								
saline	-5.59 ± 0.08	115.1 ± 24.2	-6.69 ± 0.11	68.9 ± 4.9	-7.80 ± 0.15	94.1 ± 2.9	-6.39 ± 0.29	67.3 ± 7.1
DEP	-5.53 ± 0.06	91.8 ± 4.0	-6.81 ± 0.14	77.2 ± 5.6	-7.81 ± 0.18	95.1 ± 1.9	-6.51 ± 0.27	77.4 ± 3.9
**24 h**								
saline	-5.46 ± 0.24	136.0 ± 32.4	-6.79 ± 0.18	61.2 ± 4.7	-7.64 ± 0.18	92.7 ± 2.7	-7.03 ± 0.14	41.8 ± 4.8
DEP	-5.46 ± 0.09	124.2 ± 11.4	-6.86 ± 0.14	68.3 ± 6.3	-7.81 ± 0.18	92.5 ± 2.2	-7.07 ± 0.23	58.7 ± 6.5
**Mesenteric Artery**								
**6 h**								
saline	-6.02 ± 0.15	96.1 ± 3.6	-7.29 ± 0.27	76.4 ± 7.8	**-7.82 ± 0.15**	98.1 ± 2.2	-8.19 ± 0.17	101.3 ± 0.7
DEP	-5.76 ± 0.19	99.3 ± 3.9	-7.51 ± 0.11	88.4 ± 3.6	-8.36 ± 0.05**	98.3 ± 1.4	-8.80 ± 0.24	99.6 ± 1.2
**24 h**								
saline	-6.19 ± 0.11	103.6 ± 5.9	-7.65 ± 0.23	82.0 ± 5.0	-8.40 ± 0.15	99.1 ± 1.1	-8.74 ± 0.16	102.4 ± 0.9
DEP	-6.07 ± 0.11	132.9 ± 13.1	-7.58 ± 0.17	80.6 ± 5.8	-8.28 ± 0.19	99.3 ± 3.4	-8.32 ± 0.21	102.5 ± 0.4

Results are mean ± SEM (n = 5-10), obtained by non-linear regression

*P < 0.05, **P < 0.01, control vs DEP, unpaired t-test.

EC_50 _- concentration producing 50% of maximum contraction

E_max _- maximum contraction

IC_50 _- concentration producing 50% of maximum vasodilatation

I_max _- maximum vasodilatation

To assess the possibility of the effects of DEP being apparent in blood vessels other than the thoracic aorta, vascular responses were also assessed in another conduit artery (femoral) and in a resistance artery (3^rd ^order mesenteric). Responses to phenylephrine were similar in femoral and mesenteric arteries from DEP- and saline-treated animals (*P *> 0.05; Table 3). There were no differences in the responses to ACh between saline-instilled or DEP-instilled animals in either femoral (*P *> 0.36; Figure 4e) or mesenteric arteries (*P *> 0.08; Figure 4f) at either time point. Responses to other vasodilators were similar in these vessels from DEP and saline treated animals, with the exception that there was a significantly greater sensitivity to SNP in mesenteric arteries (*P *= 0.002; two-way ANOVA; IC_50_, *P *= 0.004) from DEP-treated rats (Table 3).

## Discussion

Using a rat model, we have shown that acute pulmonary exposure to DEP induces both pulmonary and systemic inflammation, yet does not cause vascular endothelial dysfunction measured either *in vivo *or *ex vivo*.

In recent studies, we have shown both short- and long-term vascular and endothelial impairment in humans after exposure to diesel exhaust [8,9,11]. These effects appear to be mediated by the particulate components of the diesel exhaust [7,10]. To permit further investigation into the mechanism of DEP-induced vascular dysfunction, we exposed rats specifically to the particulate components of diesel exhaust via pulmonary instillation. Intratracheal instillation of particle suspensions has been shown to be a reliable way of producing excellent dispersion of particles throughout the lobes of rodent lungs and across the surface of the alveoli, leading to pulmonary effects that are directly comparable to that of inhalation studies [24-27]. Thus we used this method to test the hypothesis that DEP impairs endothelial function through the generation of pulmonary and systemic inflammation.

Inflammation has been implicated in many of the actions of DEP and other particles, including actions on the cardiovascular system [19]. It has been suggested that the pulmonary inflammation induced by particles leads to raised levels of inflammatory mediators within the circulation, that have detrimental actions on the vasculature. Pulmonary exposure to DEP produces a clear lung inflammation that peaks ~6-12 h after instillation and persists for over 24 h [18,20]. While the pulmonary actions of DEP are relatively consistent, measures of particle-induced systemic inflammatory response are notoriously variable [28-30]. In the present study we showed that DEP induced pulmonary inflammation 6 h after exposure. This was characterised by marked neutrophil infiltration, raised levels of the inflammatory mediator IL-6 and an increase in alveolar permeability, characterised by increased levels of protein in BALF. Pulmonary inflammation was largely resolved 24 h after exposure. While there was no indication of systemic inflammation at 6 h after DEP instillation, the levels of two inflammatory mediators, IL-6 and TNFα were increased in the plasma by 24 h after exposure. The time course of these responses could suggest that these factors originate from the prior inflammatory response in the lung. Previous studies in animals [31,32] and man [33,34] report raised levels of CRP in the blood following exposure to urban particulates or episodes of high air pollution, yet we did not find any changes in this mediator after DEP instillation. Differences in the type and composition of the particulate or co-pollutant may explain these discrepancies (see below). Regardless, these results highlight the need to measure multiple markers of inflammation to ensure that the systemic consequences of particle instillation are not overlooked.

In the present study we found that DEP instillation had no effect on endothelium-dependent vasodilatation, either *in vivo *or *ex vivo*. Previous studies have reported that DEP exposure by either inhalation or instillation is associated with endothelial dysfunction [35-37]. Similarly, exposure of rodents to urban PM has been reported to attenuate vascular function [38-41]. However, several studies have found that pulmonary exposure to DEP or urban PM, by both inhalation and instillation, had no effect on responses to vasodilators despite the high doses of particulate employed [42-44]. Similarly negative findings may be under-represented due to the tendency not to report negative data. The reason for these discrepancies is likely to be multifactorial, being influenced by dose and duration employed, type of particle, differences in animal species and blood vessel used as well as how the measurements are made.

The dose of particle used in the current study is high: approximately 30-fold higher than the comparable alveolar deposition from a 24 h inhalation of 100 μg/m^3 ^in man (MPPD model, adjusted rat lung surface area) [45]. Thus, we do not believe that we have failed to observe an effect due to use of an inappropriately low dose. One of the notable differences between the present instillation study and real world exposures is that here we administer the particulate alone. Gases and semi-volatile compounds in whole diesel exhaust have the potential to produce vascular effects, although data from gaseous constituents alone is highly inconsistent and often only seen at high concentrations of these pollutants [44,46]. Furthermore, in our clinical exposures, the vascular action of diesel exhaust was abolished if particles were filtered from the emissions [7,10]. A second consideration is the composition of the particulate itself. The physiochemical properties of DEP vary markedly depending on the fuel used and the engine type and running conditions. In the present study we used widely available 'standard' DEP from the National Institute of Standards and Technology (NIST), a frequently used source of DEP that acts as a benchmark for comparisons of the biological effects of DEP between studies. Interestingly isolated studies have shown that 'clean' carbon particles have the capacity to impair vascular function [47] or promote atherosclerosis [48]. Similarly, even nanoparticles believed to be inert (e.g. titanium dioxide nanoparticles) have occasional been reported to have vascular effects [41,49]. However, generally, the cardiovascular actions of these particles are small in comparison to that of urban PM or DEP [50,51], and are not evident in controlled exposures in humans [10]. Therefore, the quantities and properties of surface chemicals within DEP (e.g. organic constituents, transition metals) could account from a degree of variability between the vascular effects of different sources of DEP. Finally, species differences should also be considered, not just between human and rodent models, but also between different animal species, source of animals (e.g. inbred vs outbred animals) and the use of different animal models of disease.

Experiments in the present study were specifically designed to maximise the chances of detecting alterations in vascular function by making measurements *in vivo *where the neurohumoural systems are intact, as well as *ex vivo *both in conduit and resistance arteries. In the anaesthetised rat, both the endothelium-dependent vasodilator ACh and the endothelium-independent vasodilator SNP induced an increase in hind-limb blood flow. Both agents were delivered locally via the femoral artery to avoid reduction of systemic blood pressure and the associated reflex vasoconstriction [52], although there was a tendency for SNP to induce a small transient reduction in mean arterial blood pressure, suggesting a small degree of systemic activity. In animals that had received DEP 6 h prior to assessment of vascular function, responses to ACh in the hind-limb resistance bed were not different from those achieved in saline-instilled animals. This contrasts with our clinical studies in the forearm resistance bed, in which endothelium-dependent relaxation was impaired after diesel exhaust exposure [8,9,11]. The *in vivo *measurements were supported by a lack of effect of DEP on vascular function *ex vivo*. This lack of effect of DEP disproves our original hypothesis that pulmonary exposure to this particulate will impair vasodilator responses. However, the results do provide important insights into the mechanism of the cardiovascular effects of instilled DEP. Importantly, endothelial function was preserved at times when there was significant pulmonary (6 h) and systemic (24 h) inflammation, suggesting that inflammation alone is unlikely to account for the cardiovascular actions of inhaled particulates. Indeed, other studies have demonstrated endothelial dysfunction without inflammation [53,54], supporting the notion that the two phenomena are independent.

Endothelium-independent relaxation (SNP, isoprenaline) *ex vivo *was also unaffected by DEP instillation, suggesting that the ability of smooth muscle to relax is not impaired following exposure to DEP *in vivo*. However, in contrast, the increase in hind-limb blood flow induced by SNP *in vivo *was impaired both 6 and 24 h after administration of DEP. The change in blood flow *in vivo *depends on the balance between vasorelaxation and vasoconstriction after drug administration. The fact that the increase in hind-limb blood flow induced by SNP is reduced in animals that received DEP could, therefore, be the result of reduced vasorelaxation. A change in muscle sensitivity is unlikely as responses to ACh were not affected and we could not detect any change *ex vivo*, but we cannot rule out a change in bioactivity as a result of, say, increased oxidative stress [12,55] localised to the site of NO release from SNP. The reduced response to SNP could also be explained by increased vasoconstriction due to other counteracting constrictor influences such as endothelin or angiotensin II, or to increased sympathetic nerve activity. Alterations in these pathways could account for the raised blood pressure following PM exposure [56-58], as evidenced by the increased baseline systolic blood pressure in DEP-instilled rats in the current study. Additionally, the latter hypothesis is attractive given the evidence in the literature for increased autonomic activity and baroreceptor sensitivity in response to PM [17,59-62]. However, at present we can only speculate on the mechanism involved. Further experimentation is required to fully investigate the mechanisms involved.

## Conclusions

Using a series of *in vivo *and *ex vivo *experiments we have shown that instillation of diesel exhaust particulate in rats causes both pulmonary and systemic inflammation, but this is not associated with impaired endothelial function. However, DEP administration produced specific impairments in the response to SNP *in vivo *that require further investigation.

## Materials and methods

### Animals

Adult male Wistar rats (200-250 g; Charles River, Margate, UK; at least 4 animals per treatment group) were housed under controlled environmental conditions (21 ± 2°C; 12 h light/dark cycle) with access to tap water and standard laboratory rat chow *ad-libitum*. All rats were allowed to acclimatize to the environment for at least one week before experimental procedures were initiated. All experiments were performed according to the Animals (Scientific Procedures) Act 1986 (U.K. Home Office).

### Pulmonary instillation of DEP

DEP (SRM-2975; National Institute of Standards and Technology, Gaithersburg, USA) was suspended in 0.9% sterile saline at a stock concentration of 1 mg/mL and sonicated for 5 min (70% power; 5 Hz) in an ice bath using a probe-type sonicator (US70; Philip Harris Scientific, Lichfield, U.K.) to minimise particulate aggregation.

DEP was administered by intra-tracheal instillation as previously described [59]. Briefly, rats were anesthetised, using 5% isoflurane inhalation (Meriol, Essex, UK), and positioned head-upwards on a board inclined at a 45° angle. The vocal chords were visualised by passing a pediatric laryngoscope with a plastic cannula into the trachea. Particles (0.5 mg/rat) or vehicle (saline) were then instilled as a 0.5 mL bolus. The dose used is comparable to previous studies [20,30,63,64] and preliminary experiments showed that this dose of DEP induces systemic effects without acute toxicity [59]. An additional group of non-instilled rats was used to confirm that saline instillation itself did not have significant pulmonary and systemic actions.

### Collection and analysis of bronchoalveolar lavage fluid (BALF)

Immediately after sacrifice BALF was collected for assessment of the inflammatory responses in the lungs of instilled animals, as described [59]. Briefly, the trachea was exposed and cannulated (0.3 mm O.D., stainless steel) allowing the lungs to be lavaged with 8 mL of sterile saline. The lungs were gently aspirated and the process repeated a further three times (24 ml total). BALF was centrifuged at 1800 g for 5 min. The supernatant from the first lavage was separated into 1 mL aliquots and stored at -80°C until required for biochemical determination. Total protein levels in BALF were determined using a standard bicinchoninic acid kit (Thermo Scientific Pierce, Northumberland, UK) to assess alveolo-capillary permeability as an indicator of lung inflammation. Additionally BALF was analysed for changes in the inflammatory mediators, C-reactive protein (CRP), tumor necrosis factor alpha (TNFα) and interleukin-6 (IL-6) by enzyme-linked immunosorbent assay (ELISA; DuoSet kits, R&D Systems, Abingdon, UK) according to the manufacturer's instructions.

The cell pellets from all lavages were resuspended in 1 mL of physiological saline and combined. Total cell number was measured using an automatic cell counter (Sartorius Steadman, Chemometec, Nuclecounter^®^, Gydevang, Denmark (941-0002)). For cell differentials, cytospin preparations were performed by centrifuging (300 rpm for 3 min; Shandon cytospin 3 centrifuge) 10,000 cells in 300 μL phosphate buffered saline containing 0.1% bovine serum albumin onto Superfrost Plus glass slides (VWR International, Lutterworth, UK). Cytospins were air-dried, fixed with 100% methanol (1 min) and then stained using Diff-Quik™ (Raymond A. Lamb, London, UK). The differential cell count was performed from an average of 300 cells per slide using a hemocytometer (Sigma-Aldrich, Dorset, UK) under 400 × magnification. BALF was collected following completion of the vascular measurements under anaesthesia (see below). Data obtained for all parameters were similar to those collected previously from animals that had not undergone this surgical intervention (data not shown).

### Hematological assays

Blood was taken from the carotid artery immediately before sacrifice into ice-cooled tubes containing heparin (final concentration 100 U/mL). The mean values of different hematological parameters; white blood cells, red blood cells and platelets; were measured with the Coulter^®^A^c^.T series analyzer (Coulter Corporation, Miami, USA). The remaining blood was centrifuged at 1500 g for 5 min to isolate plasma, which was used for assessment of systemic inflammation by measuring CRP (DuoSet kit), TNFα (Quantikine kit) and IL-6 (Quantikine kit) by ELISA (R & D Systems) according to the manufacturer's instructions. Blood was collected following completion of the vascular measurements under anaesthesia (see below). Data obtained for all parameters were similar to those from animals that had not undergone this surgical intervention (data not shown).

### *In vivo *vascular function in the hind-limb

Vascular function was studied 6 or 24 h after instillation to assess changes in vascular response over the course of the DEP-induced inflammatory response. Rats were anesthetized with isoflurane (5% for induction and 2-2.5% for maintenance) and body temperature was maintained at 37°C with a thermostatically-controlled under-blanket (Homeothermic Blanket Control Unit, Harvard Apparatus, UK). The right carotid artery was exposed by blunt dissection, cannulated with a heparinised saline-filled (final concentration 100 U/mL) polyethylene tube, and connected to a fluid filled pressure transducer (Powerlab/4SP, ADInstruments, UK) for continuous monitoring of arterial blood pressure (systolic, diastolic and mean) and heart rate.

Vascular responses to drugs administrated intra-arterially were monitored in the left hind-limb based on the method of Jackson *et al*. (2010) [65]. For drug delivery the right femoral artery was exposed and a polyethylene cannula (0.61 mm o.d., 0.28 mm i.d., Portex, Jencons Scientific Ltd, Bedfordshire, UK) was inserted, gently advanced until its tip reached the bifurcation of the abdominal aorta and secured with silk ligatures at the point of insertion. For flow measurements, a midline incision was performed in the abdominal wall and the left iliac artery just distal to the aortic bifurcation was carefully isolated from surrounding tissue. A flow probe (type 0.7 V; Transonic Systems, Norfolk) was positioned around the artery and covered with surgical lubricating jelly (Fougera, Atlanta). Hind-limb blood flow (mm/min) was monitored continuously throughout the study via a Transonic TS420 flowmeter (Transonic Systems) connected to a MacLab/4e (AD instruments, AD Instruments, Sussex, UK) data acquisition system. Upon attaining a steady blood flow, boluses of the endothelium-dependent vasodilator ACh (0.07 and 0.7 μg) were delivered in a volume of 10 μL via a microsyringe (25 μL, Hamilton Bonaduz AG, Bonaduz, Switzerland) attached to the cannula leading to the abdominal aorta. Injection of 10 μL saline itself had no effect on hind-limb blood flow. ACh and saline were given in a randomised order, followed by increasing doses of the endothelium-independent, nitric oxide donor SNP (0.09 and 0.9 μg). Preliminary studies in our laboratory suggested that these doses of ACh and SNP are sufficient to increase blood flow in the hind-limb while avoiding spill-over into the systemic circulation and influencing mean blood pressure, as compared with identical doses of acetylcholine and SNP given intravenously (data not shown). Blood flow was allowed to return to baseline before administering the next drug. Femoral vascular conductance (FVC) was calculated as femoral blood flow/mean arterial pressure.

### *Ex vivo *vascular function using myography

The thoracic aorta, a region of uninjured femoral artery and 3^rd ^order mesenteric resistance arteries were isolated from animals 6 or 24 h after instillation, and cleaned of connective tissue. Segments of aorta (~5 mm length), femoral and mesenteric arteries (both 1-2 mm length) were mounted in a multi-myograph system (610 M; Danish Myo Technology, Aarhus, Denmark) [12], using 40 μm diameter wire for smaller vessels. Vessels were submerged in Krebs buffer (composition in mM: 118.4 NaCl, 25 NaHCO_3_, 11 glucose, 4.7 KCl, 1.2 MgSO_4_, 1.2 KH_2_PO_4_, 0.027 ethylenediaminetetraacetic acid, 2.5 CaCl_2_) bubbled with 5% CO_2_/95% O_2 _at 37°C before a baseline tension of 14.7 mN (aorta), 8 mN (femoral) or 4 mN (mesenteric) was gradually applied over 10 min and vessels were allowed to equilibrate for a further 30 min. Preliminary experiments showed that these pretension levels produced optimal contraction and dilatation responses. Data from force transducers were processed by a MacLab/4e analogue-digital converter displayed through Chart™ software (AD Instruments, Sussex, UK).

Vessel viability was confirmed by a contractile response on addition of 80 mM KCl, repeated 3 times (aorta) or serial addition of high K^+^Krebs (Krebs with substitution of 4.7 mM NaCl and 118.4 mM KCl) together with 10 μM noradrenaline (femoral and mesenteric arteries). Concentration-response curves to phenylephrine (PE; 1 nM - 10 μM) were obtained and a concentration that produced 80% maximum contraction (EC_80_; 0.1-1 μM) was chosen for each individual arterial segment. Following sub-maximal contraction with the appropriate concentration of PE, cumulative concentration-response curves were obtained for acetylcholine (1 nM - 10 μM), SNP (0.1 nM - 3 μM) and a nitric oxide-independent vasodilator (isoprenaline; 0.1 nM - 10 μM). At least 30 min washout was allowed before application of subsequent drugs.

Blood vessels were isolated from taken from regions undamaged by the surgical procedure, from animals that had undergone vascular measurements *in vivo*. To obtain sufficient n-numbers for all vessel types, blood vessels were also obtained from additional animals that had not undergone the surgical procedure. Responses were identical in both groups of animals, therefore, the data-sets were combined.

### Drugs and reagents

Krebs salts were obtained from VWR International (Lutterworth, UK). All other drugs were obtained from Sigma Ltd. (Poole, UK). All drugs were dissolved and diluted in saline or Krebs buffer. Preliminary experiments confirmed that, in all cases, addition of vehicle alone had no effect on any of the parameters measured.

### Statistical analysis

Data are expressed as the mean ± standard error of the mean (SEM). Differences in FVC response were expressed as a change from baseline. Myography responses are expressed as percentage of the pre-contraction to EC_80 _PE, where positive values represent vasodilatation and 100% vasodilatation represents a complete abolition of PE-induced tone. Statistical comparisons were performed by one-way analysis of variance (ANOVA) followed by Tukey's post-hoc tests (pulmonary and systemic inflammation), two-way ANOVA using the Bonferroni post-hoc test (blood flow and blood pressure, *ex vivo *concentration response curves) or unpaired Student's t-test (*ex vivo *EC_50 _and Emax values), unless otherwise stated. Statistical analyses were performed using GraphPad Prism software (V5.0; GraphPad Software Inc, USA). P < 0.05 was considered to be statistically significant.

## Abbreviations

ACh: acetylcholine; ANOVA: analysis of variance; BALF: bronchoalveolar lavage fluid; CRP: C-reactive protein; DEP: diesel exhaust particulate; FVC: femoral vascular conductance; HBF: hind-limb blood flow; IL-6: Interleukin 6; NIST: National Institute of Standards and Technology; PE: phenylephrine; PM: particulate matter; SEM: standard error of the mean; SNP: sodium nitroprusside; TNFα: tumour necrosis factor alpha.

## Competing interests

The authors declare that they have no competing interests.

## Authors' contributions

SR carried out the *in vivo *vascular studies and measures of inflammation, performed the statistical analysis and drafted the manuscript. GAG conceived the *in vivo *study, participated in its design and coordination, helped to draft the manuscript. RD assisted with the particle instillations and design of the pulmonary assays. SGM carried out the aortic *ex vivo *vascular studies. PWFH participated in the design of the study, the surgical procedures and helped to draft the manuscript. CAS and DEN helped to draft the manuscript. MRM carried out the femoral and mesenteric *ex vivo *vascular studies and statistical analysis, participated in its design and coordination and helped to draft the manuscript. All authors read and approved the final manuscript.
